# A Microencapsulated Mixture of Eugenol and Garlic Tincture Supplementation Mitigates the Effect of Necrotic Enteritis on Intestinal Integrity and Increases Goblet Cells in Broilers

**DOI:** 10.3390/microorganisms9071451

**Published:** 2021-07-06

**Authors:** Alip Kumar, Sarbast K. Kheravii, Catherine Ionescu, Alexandra Blanchard, Reza Barekatain, Yadav S. Bajagai, Shu-Biao Wu

**Affiliations:** 1 School of Environmental and Rural Science, University of New England, Armidale, NSW 2351, Australia; akumar26@myune.edu.au (A.K.); sqassim2@une.edu.au (S.K.K.); 2 ADMi|Pancosma SA, A-One Business Center, La Piece 3, CH-1180 Rolle, Switzerland; Catherine.Ionescu@pancosma.com (C.I.); Alexandra.Blanchard@pancosma.com (A.B.); 3 South Australian Research and Development Institute, Roseworthy Campus, University of Adelaide, Roseworthy, SA 5371, Australia; Reza.Barekatain@sa.gov.au; 4 Institute for Future Farming Systems, Central Queensland University, Rockhampton, QLD 4702, Australia; y.sharmabajagai@cqu.edu.au

**Keywords:** plant extract, intestinal health, alternative to antimicrobials, clinical necrotic enteritis, broiler chicken

## Abstract

This study was conducted to examine the effects of a plant extract mixture, a microencapsulated product composed of eugenol and garlic tincture (PE), on intestinal health in broilers under necrotic enteritis (NE) challenge. A total of 960 d-old mixed-sex Cobb 500 chicks were randomly distributed to 48-floor pens housing 20 birds per pen. Six treatments were applied: UC, unchallenged control; CC, challenged control; PE, challenged group plus PE; AM, challenged group plus antimicrobial (AM); FAP, challenged group plus a full dose of AM with PE; HAP, challenged group plus a half dose of AM with PE in starter, grower and finisher phases. Birds in the challenged groups were inoculated with *Eimeria* spp. on d 9 and *Clostridium* *perfringens* on d 14. On d 16, the CC group had increased serum fluorescein isothiocyanate dextran (FITC-d), reduced villus surface area, goblet cell number, upregulated *CLDN1*, *JAM2* genes and reduced microbial diversity compared to the UC group (*p* < 0.05). Birds fed PE had reduced FITC-d, increased goblet cell number and *Bifidobacterium* compared to the CC group (*p* < 0.05). Birds fed PE had reduced *CLDN5* expression in male birds, and *Bacteroides* spp. in female birds than CC group (*p* < 0.05). These findings suggest that PE supplementation mitigates the effect of NE by improving the intestinal health of birds.

## 1. Introduction

Necrotic enteritis (NE) is a devastating enteric bacterial disease in the highly productive poultry industry with an estimated profitability loss of over US$6 billion per annum [1]. It is primarily caused by *Clostridium perfringens*, a Gram-positive, ubiquitous, anaerobic, spore-forming bacterium that is present in the normal intestinal flora of healthy chickens [2]. The bacterium *C. perfringens* in the intestinal tract under the favourable conditions together with one or more predisposing factors can become pathogenic due to the overgrowth of NetB toxin-producing strains leading to the occurrence of NE. Among many, coccidiosis and a high level of animal protein are the most important predisposing factors that were extensively studied [3,4]. Besides, other factors including a high level of non-starch polysaccharides in feed, intestinal pH change, high stocking density, contaminated litter or feed, and poor hygienic environment can compromise the intestinal health of birds and create a favourable environment for the proliferation of *C. perfringens* and the subsequent development of NE [5,6]. The sub-clinical form of NE is characterised by impaired performance and reduced feed efficiency, whereas the clinical form of NE is manifested by high flock mortality of up to 50% [7,8]. In addition, NE damages intestinal mucosa and barrier functions by altering the expression of genes coding tight junction proteins. It also changes the microbial community balance and reduces diversity which leads to a disruption of balanced microbial inhabitants [9,10,11].

Traditionally, in-feed antimicrobials (AM) have been used as growth promoters, which are effective against NE. However, the ban and/or phasing-out of in-feed AM from the poultry industry, owing to increasing public health concern about the development of AM-resistant bacteria, has led to the increased incidence of enteric diseases such as NE [12,13]. This has led to increased interest in exploring potential alternatives to in-feed AM that can effectively control enteric diseases such as NE.

Plant extracts and their bioactive compounds have been widely used in traditional medicine for decades [14]. In recent years, plant extracts have gained increased attention in animal nutrition, mainly due to their beneficial effects on performance and health [15,16]. Promising effects of the plant extracts have been shown to mitigate the negative effects of NE in broilers under challenged conditions [16,17]. Bioactive compounds derived from plants, such as herbs and spices, are known to have antimicrobial, antifungal, antiparasitic, anti-inflammatory, and antioxidative properties [18,19]. Studies have also shown that the inclusion of plant extracts can modulate microbiota composition and structure by increasing beneficial bacteria and decreasing pathogenic bacterial loads [16,20]. It has shown that plant extracts supplementation can increase immune cells, improve intestinal integrity, and reduce oxidative stress through antioxidative and immunomodulatory effects [19,21,22,23]. Among many potential plant extracts and their bioactive compounds, the supplementations of garlic and eugenol alone or in combination with different plant extracts have shown positive effects on growth performance and intestinal health [24,25,26,27,28]. A recent study reported positive effects of a microencapsulated product composed of eugenol and garlic tincture on performance under a subclinical NE [29]. However, the mode of action of plant extracts in mitigating NE effects on intestinal health is not well-documented. Moreover, the efficacy of plant extracts as alternatives to in-feed AM in broilers under severe diseased conditions has been less known and necessitates further investigation.

It was hypothesised that the supplementation of plant extract, a microencapsulated product composed of eugenol and garlic tincture (PE), may help mitigate the negative effects of clinical NE on intestinal health. This study was designed to examine the capability of PE to improve intestinal health status under severe NE challenge. The underlying mechanism of action of PE in controlling NE was investigated by determining the intestinal integrity, duodenal histomorphology, jejunal gene expressions and ileal and caecal microbiota composition in NE challenged broilers. The potential of PE in the mitigation of clinical NE effects on intestinal health was compared against an AM agent. In addition, it was hypothesised that the supplementation of PE in combination with AM may exert synergistic effects in improving intestinal health parameters. Therefore, the current study was also designed to examine the effects of PE in combination with full and half dosages of AM in broilers under clinical NE challenge and to compare their effectiveness with the supplementation of an AM alone.

## 2. Materials and Methods

### 2.1. Ethics Statement

The study was reviewed and approved by the Animal Ethics Committee of University of New England, Armidale, NSW 2351, Australia (AEC18-116). The study followed all the regulations for the use of animals for scientific purposes assigned by the Australian Bureau of Animal Health [30].

### 2.2. Design and Husbandry

A total of 288 mixed-sex Cobb 500 birds were sampled from 960 birds used for a performance experiment [31]. The birds were obtained on hatching day from Baiada hatchery in Tamworth, NSW, Australia. All the birds were vaccinated against diseases such as infectious bronchitis (spray, MSD Animal Health, Kenilworth, NJ 07033, USA) and Newcastle disease (spray, Zoetis, Parsippany, NJ 07054, USA) at the hatchery. Upon arrival, the birds were weighed and randomly allocated to 48-floor pens measuring 75 × 120 cm^2^ in a completely randomised design (CRD). The gender of birds was determined at an earlier age by feather DNA sexing using high-resolution melting curve analysis [32]. The birds were raised in a climate-controlled house with softwood shavings as bedding materials to a depth of 8 cm. Each pen was featured a tube feeder and 3 nipple drinkers providing ad libitum feed and freshwater. Lighting, temperature and relative humidity were maintained following Cobb 500 guidelines [33].

### 2.3. Dietary Treatments

Six treatment groups each with 8 replicate pens were applied in this study and each pen housed 20 birds as an experimental unit among which randomly chosen 6 birds were sampled for the relevant analysis. The six treatment groups were comprised of one unchallenged group as control and 5 challenged groups to examine the effects of PE, a microencapsulated product composed of 10% eugenol and 10% garlic tincture on the intestinal health of broilers under clinical NE challenge as shown in Table 1. The treatments were: UC, unchallenged control, without additive or in-feed antimicrobial (AM); CC, NE challenged control, without additive or in-feed AM; PE, NE challenged group plus additive PE at 100 part per million (ppm); AM, NE challenged group plus AM containing 50 ppm each active compound of narasin and nicarbazin; FAP, NE challenged group plus a full dose of AM with PE; HAP, NE challenged group plus a half dose of AM with PE in starter, grower and finisher phases. Diets were based on wheat, soybean meal, sorghum, and meat and bone meal. Diets were formulated considering nutrients and the matrix values of additives and phytase to meet the nutrient requirements of Cobb 500 birds [34]. Prior to feed formulation, the nutrient contents of feed ingredients were determined using near-infrared spectroscopy (NIRS, Evonik AminoProx, Essen, Germany). Diets were cold-pelleted and fed in 3 phases: starter phase (d 0 to 9), grower phase (d 9 to 21) and finisher phase (d 21 to 35). Starter feed was crumbled further to maximise feed intake. The detail of diet composition for each phase was reported earlier [31].

### 2.4. Necrotic Enteritis Challenge

The NE challenge model was applied in the present study following previously reported challenge protocols [4,35] where field strains of *Eimeria* spp. oocysts were employed as a predisposing factor and *C. perfringens* as the causative agent to introduce NE. In brief, on d 9, challenged birds were orally gavaged by using crop needles with field strains of *Eimeria* spp. containing 5000 sporulated oocysts of both *E*. *acervulina* and *E*. *maxima*, and 2500 sporulated oocysts of *E*. *brunetti* in 1 mL of 1% (*w/v*) sterile phosphate-buffered saline (PBS) (Eimeria Pty Ltd., Ringwood, VIC, Australia). On d 14, challenged birds were orally gavaged by using crop needles with 1 mL of *C. perfringens* (EHE-NE18) containing approximately 10^8^ CFU (CSIRO Livestock, Geelong, VIC, Australia). Simultaneously, unchallenged birds were orally gavaged by using crop needles with 1 mL PBS on d 9 and sterile medium on d 14.

### 2.5. Sampling and FITC-d Inoculation

On d 8, 2 randomly chosen birds (1 male and 1 female) from each pen were weighed, electrically stunned (JF poultry equipment, Weltevreden Park, South Africa) and euthanised by cervical dislocation to collect duodenal tissue samples for histomorphology and jejunal tissue samples for gene expressions.

On d 16, 4 birds (2 males and 2 females) from each pen were randomly chosen, weighed and orally gavaged with 1 mL fluorescein isothiocyanate dextran (FITC-d; average molecular weight: 4000, Sigma–Aldrich Co., St. Louis, MO, USA) containing 4.17 mg/kg body weight on average. The inoculated birds were stunned after 2.5 h (approximately) of inoculation by an electric stunner (JF poultry equipment, Weltevreden Park, South Africa) and euthanised by cervical dislocation and followed by decapitation to collect blood, and intestinal samples. Ileal and caecal contents from 2 males and 2 females per pen were collected separately in 2 mL Eppendorf tubes and stored at −20 °C for microbiota analysis. Approximately 2 cm of the proximal jejunal tissue from 2 males and 2 females per pen was excised, flushed with chilled PBS and collected in 2 mL Eppendorf tubes containing RNA later (Invitrogen, Thermo Fisher Scientific, Carlsbad, CA, USA) and kept at 4 °C for 4 h before stored in −20 °C for gene expression analysis. Proximal duodenal tissue from 2 males was excised, flushed with chilled PBS, fixed in 10% buffered formalin and kept in containers containing formalin until samples were processed for histology measurements.

Blood samples from 4 sampled birds per pen were collected separately in a clot activator Vacutainer tube from the jugular vein by decapitation method. Blood samples were kept at room temperature for approximately 3 h to allow clotting, centrifuged at 3000× *g* for 10 min to separate serum samples and immediately stored in a −20 °C until the measurements were performed.

### 2.6. Serum FITC-d Measurement

Fluorescence concentrations of diluted serum (1:1 in PBS) were determined at the excitation wavelength of 485 nm and an emission wavelength of 528 nm on a multi-mode microplate reader (SpectraMax M2e, Molecular Devices, San Jose, CA, USA) and FITC-d concentration per mL of serum was calculated based on a standard curve constructed with a known concentration of FITC-d and expressed as µg/mL corrected with the individual body weight of the birds.

### 2.7. Histomorphology

Proximal duodenal tissues collected from male birds only for intestinal morphology were sectioned (4 μm) and processed using standard Haematoxylin and Eosin assay as described by [36]. Villus height (VH), crypt depth (CD) and villus width (VW) were measured with a minimum of 25 villi and associated crypts randomly chosen for measurements. A previously described formula [37] was used to calculate villus surface area (VSA); VSA = 2π (VW/2) (VH). Periodic acid–Schiff staining was used to visualise goblet cells. Goblet cell numbers were counted with a minimum of 20 villi and associated crypts randomly chosen for measurements and expressed as goblet cell numbers per villus. Slides were scanned and parameters were measured using NDP.view 2.5 software (Hamamatsu Photonics K.K., Higashi-ku, Hamamatsu city, 431-3196, Japan).

### 2.8. RNA Extraction and cDNA Synthesis

Total RNA from each jejunal tissue sample collected on d 8 and d 16 was extracted after homogenisation in TRIsure^TM^ (Bioline, Sydney, NSW, Australia) according to the manufacturer’s instructions and following the method described by Samiullah et al. [38] with slight modifications. Approximately 60 mg of jejunal tissue samples were placed with a 3 mm metal bead in a 2 mL Eppendorf tube. Then, 1 mL TRIsure was added to the Eppendorf tube and homogenised well using IKA homogeniser. After homogenisation, the samples were incubated for 5 min at room temperature. After incubation, 200 μL of chloroform was added and shaken vigorously by hand for 15 s and followed by incubation for 3 min at room temperature. The samples were centrifuged at 12,000× *g* for 15 min at 4 °C. The aqueous phase of the samples was transferred (upper phase) very carefully into a 1.5 mL tube. A volume of 0.5 mL chilled isopropyl alcohol was added and shaken vigorously by hand and followed by incubation for 10 min at room temperature. The samples were centrifuged for 10 min at 12,000× *g* at 4 °C. The RNA pellet was visible at this stage. All the supernatant alcohol was removed, and 1 mL of 75% ethanol was added to wash the pellet. The samples were vortexed and centrifuged at 7500× *g* for 5 min at 4 °C. Again, all the supernatant alcohol was removed. Then the RNA pellet was re-dissolved in 150 μL of Nuclease free water and pipetted the solution up and down until the pellet was completely dissolved in the water. The extracted RNA samples were stored at −80 °C. The extracted RNA samples were purified using Rneasy Mini Kit, (Qiagen, Hilden, Germany) based on the manufacturer’s instructions. Approximately 100 μL of extracted RNA samples were taken in 2 mL Eppendorf tube and 0.6 mL of the Lysis buffer RLY-ethanol premix (1:1 ratio) was added. The samples were then mixed by vortexing. Then, the ISOLATE II filter (violet) was placed in a collection tube and the lysate (RNA samples with Lysis buffer RLY-ethanol premix) was loaded in the filter and followed by centrifugation at 11,000× *g* for 1 min. The ISOLATE II filter was discarded. A volume of 350 μL 70% ethanol was added to homogenise the lysate. The samples were pipetted up and down (5 times) for mixing properly. Then, the lysate was pipetted 2–3 times and loaded to the newly placed ISOLATE II RNA mini-column (blue) in a 2 mL collection tube and centrifuged at 11,000× *g* for 30 s. The column was placed in a new 2 mL collection tube. A volume of 350 μL Membrane Desalting Buffer (MEM) was added and centrifuged at 11,000× *g* for 1 min to dry the membrane. After that, 100 μL of DNase I (RDN) reaction mixture was prepared by adding 10μL reconstituted DNase I to 90 μL Reaction buffer and mixed the mixture by flicking, gently. A volume of 95 μL DNase I reaction mixture was added directly onto the centre of the silica membrane and followed by incubation for 15 min at room temperature. Then, the samples were washed 3 times using wash buffer (200 μL RW1, 600 μL RW2 and 250 μL RW2) and centrifuged each time with a new collection tube to dry the cilica membrane. After that, a volume of 100 μL of Nuclease-free water was added directly onto center of the silica membrane and followed by centrifugation at 11,000× *g* for 1 min. The column was discarded, and the purified RNA sample was stored at −80 °C. The quantity and purity of the total RNA samples were measured using a NanoDrop ND-8000 spectrophotometer (Thermo Fisher Scientific, Waltham, MA, USA). An RNA 6000 Nano kit was applied to determine RNA integrity number (RIN) using the Agilent 2100 Bioanalyzer (Agilent Technologies, Inc., Waldbronn, Germany). The purified RNA samples were considered as high-quality if the value of 260/230 was higher than 1.8, 260/280 value between 2.0 to 2.2, and the RIN number was greater than 7.0. The isolated RNA of the tissue sample was reverse transcribed using the QuantiTect Reverse Transcription Kit (Qiagen, Hilden, Germany) according to the manufacturer’s instructions. In brief, 1 µg of each total RNA sample was incubated at 42 °C for 2 min in 2 µL of 7× genomic DNA (gDNA) Wipeout Buffer to avoid gDNA contamination. After that, the gDNA elimination reaction was added to reverse-transcription reaction components contained one µL of Quantiscript Reverse Transcriptase, 4 µL of 7× Quantiscript RT Buffer, and one µL of RT Primer Mix and mixed appropriately. The Rotorgene 6000 real-time PCR machine (Corbett, Sydney, NSW, Australia) was applied to incubate the mixture at 42 °C for 15 min followed by 95 °C for 3 min to convert the RNA into cDNA. The cDNA samples were then diluted 10 times with Nuclease-free water and kept at −20 °C for further analysis.

### 2.9. Real-Time Quantitative Polymerase Chain Reaction (RT-qPCR)

Amplification and detection were performed in duplicates using an SYBR Green kit SensiFAST™ SYBR^®^ No-ROX (Bioline, Sydney, Australia) with Rotorgene 6000 real-time PCR machine (Corbett Research, Sydney, NSW, Australia). The PCR reaction was carried out in a volume of 10 µL containing 2 µL of 10× diluted cDNA template, 400 mm of each primer, and 5 µL of 2× SensiFAST™ SYBR^®^ No-ROX. A total of 8 house-keeping genes, namely, GAPDH, YWHAZ, 18S, ACTB, HMBS, HPRT1, SDHA, and TBP were used for the optimisation of reference genes using the gene expression stability measure (geNorm M) module in qbase+ software version 3.0 (Biogazelle, Zwijnbeke, Belgium). The two most stable house-keeping genes with the lowest M-value (<0.5), TBP (M-value = 0.383) and YWHAZ (M-value = 0.383) for d 8, and GAPDH (M-value = 0.085) and TBP (M-value = 0.088) for d 16 were chosen as optimised reference genes to normalise the expression of the target genes. The amplification cycle (Cq) values for candidate target genes were collected and imported into qBase+ version 3.0 software (Biogazelle, Zwijnbeke, Belgium) and analysed against the reference genes. The qBase+ employed the arithmetic mean method to transform logarithmic Cq values to linear relative quantity applying the exponential function for relative quantification of genes [39,40] and the output data was exported for the statistical analysis. The normalised relative quantities (NRQ) values were calculated and analysed across all samples for each target gene. The primers employed in this study were either sourced from previously published studies as presented in Table 2. Agilent 2100 Bioanalyzer (Agilent Technologies, Inc., Waldron, Germany) was used to determine the specificity of each primer pair prior to qPCR analysis using Agilent DNA 1000 Kit (Agilent Technologies, Inc., Waldron, Germany), and only specific primers amplifying target fragments were used in the qPCR assay.

### 2.10. Extraction of Ileal Bacterial DNA

The DNA from frozen ileal digesta samples collected on d 16 was extracted using a QIAamp DNA Stool Mini Kit, Catalogue No. 51,504 (Qiagen, Inc., Hilden, Germany) following the method described by Kheravii et al. [48].

### 2.11. Extraction of Cecal Bacterial DNA

The DNA of frozen caecal digesta samples collected on d 16 was extracted using PowerFecal QIAcube^®^ HT Kit (Qiagen, Inc., Hilden, Germany) following the method described by Kumar et al. [49].

### 2.12. Quantification of Ileal and Caecal Bacterial DNA

The bacterial DNA quantification methods described previously [50,51] were employed in the current study. Briefly, the extracted ileal and caecal digesta DNA were diluted 20 times in nuclease-free water, and 8 major bacterial groups were quantified through quantitative real-time PCR with Rotorgene 6000 (Corbett, Qiagen, Inc., Hilden, Germany). The master mix containing SYBR-Green (SensiMix SYBR No-Rox, Bioline, TN, USA) was used for duplicated qPCR reactions for each sample. The reaction in a total volume of 10 μL containing 2 μL of diluted caecal DNA, 300 mmol/L of forward and reverse primers, and 5 μL of 2× SensiMix. The reaction mix containing SYBRGreen was used for the quantification of genomic DNA copies of *Lactobacillus* spp., *Bifidobacterium* spp., *Bacteroides* spp., *Bacillus* spp., *Ruminococcus* spp., *Enterobacteriaceae*, total anaerobic bacteria. SensiFAST Probe SYBR No-ROX (Bioline, TN, USA) was used for *C. perfringens* for the Taqman-based assay. The specific 16S rRNA primers applied for quantifying these bacterial groups are shown in Table 3. The number of target DNA copies was calculated, and the bacterial quantity was expressed as log_10_ (genomic DNA copy number)/g digesta.

### 2.13. 16S rRNA Gene Sequencing and Data Analysis

The V3-V4 regions of 16S rRNA genes were sequenced using Illumina MiSeq 2× 300 bp paired-end sequencing with 341f and 805r primers. The quality of the sequence reads was checked with fastQC v0.11.9 (Babraham Institute, Cambridge, UK) [60]. The upstream analysis of the sequence was performed with QIIME2 (https://qiime2.org/ (accessed on 29 April 2021)) [61] by employing DADA2 [62] plugin for quality control and denoising. The downstream statistical analysis of the amplicon sequence variant (ASV) matrix and visualisation was performed with Calypso (http://cgenome.net:8080/calypso-8.84 (accessed on 29 April 2021)) [63]. Experimental treatments and sex were set as biological condition and secondary group, respectively. Two-way ANOVA analysis was performed for the univariate analysis in Calypso.

### 2.14. Data Analysis

All data generated in this study were examined for normal distribution before statistical analysis. The histomorphology data were analysed as a completely randomised design using Fit Model of JMP^®^ 14.0 (SAS Institute, Cary, NC, USA). The significant differences between means were separated by the Least significant difference test. Data collected from both male and female birds were subjected to 2-way ANOVA analysis as a 6 × 2 factorial arrangement to assess the main effects of the experimental treatment and sex, and interaction of treatment × sex. The means were declared significantly different at *p* < 0.05.

## 3. Results

### 3.1. Serum FITC-d Concentration

The effects of NE challenge and PE on serum FITC-d in broilers are shown in Table 4. Two-way ANOVA analysis indicated the main effect of experimental treatments was significant on serum FITC-d concentration on d 16 (*p* < 0.001). The sex effect was not significant (*p* = 0.064) and there was no interaction between experimental treatment and sex (*p* = 0.502). The NE challenge significantly increased serum FITC-d concentration in the CC group compared to the UC group. The supplementation of PE reduced serum FITC-d concentration compared to the CC group, but increased it compared to the AM group. Birds fed FAP had similar but fed HAP had higher serum FITC-d concentration compared to the AM group.

### 3.2. Histomorphology and Goblet Cell Number

The effects of NE challenge and PE on duodenum histology on d 8 and d 16 are presented in Table 5. One-way ANOVA analysis indicated that goblet cell number per villus on d 8 (*p* = 0.041) showed significant differences. Prior to the NE challenge (d 8), goblet cell numbers per villus were not different between UC and CC groups as expected. Birds fed PE had similar goblet cell numbers per villus compared to the CC and AM groups. However, birds fed FAP had higher goblet cell numbers per villus compared to UC and CC while not significantly different from the rest of the additive groups.

One-way ANOVA analysis indicated that VH (*p* < 0.001), CD (*p* < 0.001), VH:CD (*p* < 0.001), VSA (*p* < 0.001) and goblet cell number per villus (*p* < 0.001) on d 16 showed significant differences. The NE challenge without supplementations significantly decreased VH, VH:CD, VSA, goblet cell number per villus and increased CD and VW. Birds supplemented with PE had increased goblet cell numbers per villus compared to the CC group but VW, VH, CD, VH:CD and VSA were not different. Compared to the AM group, birds fed PE had lower VH, CD and VSA whereas no differences were observed for VW, VH:CD and goblet cell numbers per villus. Birds fed AM, FAP and HAP had similar VH, CD, VH:CD VSA and goblet cell numbers per villus, while FAP group had higher VW than HAP.

### 3.3. Jejunal Gene Expression on d 8

The effects of experimental treatment and sex on jejunal gene expressions on d 8 are presented in Table 6. Two-way ANOVA analysis indicated a significant main effect of sex on *OCLN* (*p* = 0.033) and *TJP1* (*p* = 0.026), where female birds had a lower expression of *OCLN* and higher expression of *TJP1* genes compared to the male birds. The expression of *CLDN1* and *MUC2* genes were not different between male and female birds. Experimental treatments had no effects on *CLDN1*, *MUC2*, *OCLN* and *TJP1* genes, and no interaction between experimental treatment and sex was observed on these genes (*p* > 0.05) on d 8.

### 3.4. Jejunal Gene Expression on d 16

The effects of experimental treatment and sex on jejunal gene expressions on d 16 are shown in Table 7. Two-way ANOVA analysis demonstrated significant effects of experimental treatment on *CLDN1* (*p* < 0.001) and *JAM2* (*p* < 0.001) gene expression and sex on *TJP1* (*p* = 0.038) gene expression. There was no interaction between experimental treatment and sex (*p* > 0.05).

The NE challenge without supplementations upregulated the expression of *CLDN1* and *JAM2* genes, but not *TJP1*. The expression of *CLDN1* gene was not different among the challenged groups with or without additives supplementation. The expression of *JAM2* was not different between PE and CC groups but higher in the PE supplemented birds compared to the AM group. The expression of *JAM2* was not different between FAP and AM groups but higher in the HAP group compared to the AM group. The gene *TJP1* was downregulated in female birds compared to the male birds but the expression of *CLDN1* and *JAM2* genes were not different between male and female birds on d 16.

The expression of genes *CLDN5*, *OCLN* and *MUC2* is shown in Table 8. Two-way ANOVA analysis showed that there was an interaction between experimental treatment and sex on the expression of *CLDN5* (*p* = 0.004), *OCLN* (*p* < 0.001) and *MUC2* (*p* = 0.029) genes. Birds fed PE, AM and FAP had downregulated *CLDN5* gene compared to the CC group in male birds but not in the female birds. Also, the UC and CC male birds had higher expression of *CLDN5* than the counterpart female birds, while FAP male birds had lower expression than the counterpart female birds. The challenged groups with or without additives supplementation had a downregulated *OCLN* gene compared to the UC group in the male birds, but no difference was observed in female birds. Birds in the CC group had reduced *MUC2* expression compared to the UC group, but the extent of reduction differed between males and females with the male birds responded to a greater extent. Birds fed FAP and HAP had upregulated *MUC2* gene compared to the CC group in male birds but not in female birds.

### 3.5. Ileal Bacterial Load by qPCR

The main effects of experimental treatment and sex on ileal microbiota on d 16 are shown in Table 9. Two-way ANOVA analysis demonstrated significant main effects of the experimental treatment on *Lactobacillus* spp. (*p* < 0.001) *Bifidobacteria* spp. (*p* < 0.001), total bacteria (*p* < 0.001), *Bacillus* spp. (*p* < 0.001), *Enterobacteriaceae* (*p* < 0.001) and *C. perfringens* (*p* < 0.001), and sex on *Lactobacillus* spp. (*p* = 0.008), *Bacillus* spp. (*p* = 0.039) and *Ruminococcus* spp. (*p* < 0.001). There was no interaction between experimental treatments and sex (*p* > 0.05) except for *Bacteroides* spp. (*p* = 0.044).

**Figure 1:**
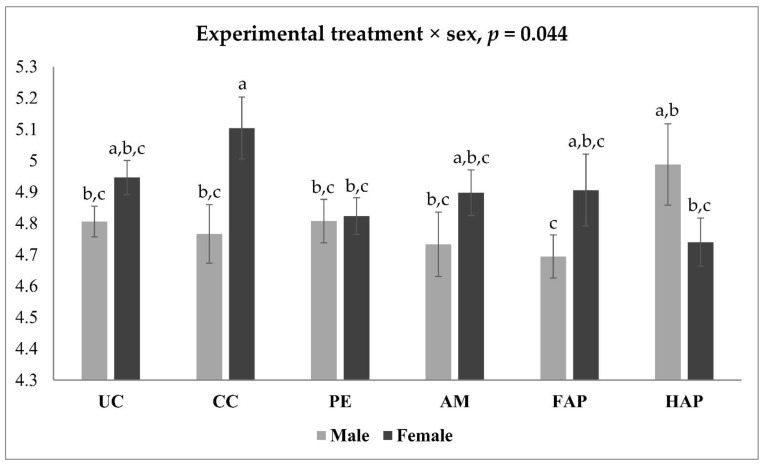
Interaction between experimental treatment and sex on Bacteroides spp. loads on d 16 in ileal content. UC, unchallenged control; CC, challenged control; PE, plant extract; AM, antimicrobial (narasin and nicarbazin; 50 ppm each active compound); FAP, full dose of AM plus PE; HAP, half dose of AM plus PE. Challenged birds were orally gavaged with Eimeria spp. on d 9 and Clostridium perfringens on d 14. ^a–c^ Mean values with no common superscripts differ significantly (*p* < 0.05).

The NE challenge significantly increased *Lactobacillus* spp. and *Bifidobacteria* spp., *Bacillus* spp., *Enterobacteriaceae*, *C. perfringens* and total bacteria loads in the CC group compared to the UC group. Birds fed PE had similar *Lactobacillus* spp. and *Bifidobacteria* spp., *Bacillus* spp., *Enterobacteriaceae*, *C. perfringens* and total bacteria loads compared to the CC group, and had higher loads compared to the AM group. Birds fed FAP and HAP had similar *Lactobacillus* spp. and *Bifidobacteria* spp., *Bacillus* spp., *Enterobacteriaceae* and total bacteria loads whereas the HAP group had higher *C. perfringens* loads compared to the AM group. Female birds had increased *Lactobacillus* spp., *Bifidobacteria* spp. and *Ruminococcus* spp. compared to the male birds. The interaction between experimental treatment and sex on *Bacteroides* spp. in the ileal content is shown in Figure 1. Birds fed PE and HAP had decreased *Bacteroides* spp. loads compared to the CC group in female birds but not in male birds.

### 3.6. Caecal Bacterial Load by qPCR

The effects of experimental treatment and sex on caecal microbiota on d 16 are shown in Table 10. Two-way ANOVA analysis showed significant main effects of experimental treatment on *Lactobacillus* spp. (*p* < 0.001), *Bifidobacteria* spp. (*p* = 0.007), total bacteria (*p* = 0.018), *Bacillus* spp. (*p* < 0.001), *Ruminococcus* spp. (*p* < 0.001), *Enterobacteriaceae* (*p* < 0.001) and *C. perfringens* (*p* < 0.001), and sex on *Lactobacillus* spp. (*p* < 0.001), *Bifidobacteria* spp. (*p* = 0.007), *Ruminococcus* spp. (*p* = 0.031) and total bacteria (*p* = 0.007). There was no interaction between experimental treatments and sex (*p* > 0.05).

The NE challenge significantly increased *Lactobacillus* spp., *Bifidobacteria* spp., *Enterobacteriaceae*, *C. perfringens*, total bacteria and decreased *Bacillus* spp. and *Ruminococcus* spp. loads (CC vs. UC). Birds fed PE had decreased *Lactobacillus* spp. load compared to the CC group but *Bifidobacteria* spp., total bacteria, *Bacillus* spp., *Ruminococcus* spp., *Enterobacteriaceae, C*. *perfringens* loads were not different. Birds fed PE had similar *Bifidobacteria* spp., *Bacillus* spp., *Ruminococcus* spp., total bacteria but had higher *Lactobacillus* spp., *Enterobacteriaceae* and *C. perfringens* loads compared to the AM group. Birds fed FAP and HAP had similar *Lactobacillus* spp., *Bifidobacteria* spp., *Bacillus* spp., *Ruminococcus* spp. *Enterobacteriaceae* and total bacteria loads whereas the HAP group had increased *C. perfringens* loads compared to the AM group. Female birds had increased *Lactobacillus* spp., *Bifidobacteria* spp., total bacteria and decreased *Ruminococcus* spp. compared to the male birds.

### 3.7. Caecal Microbiota Diversity

**Figure 2:**
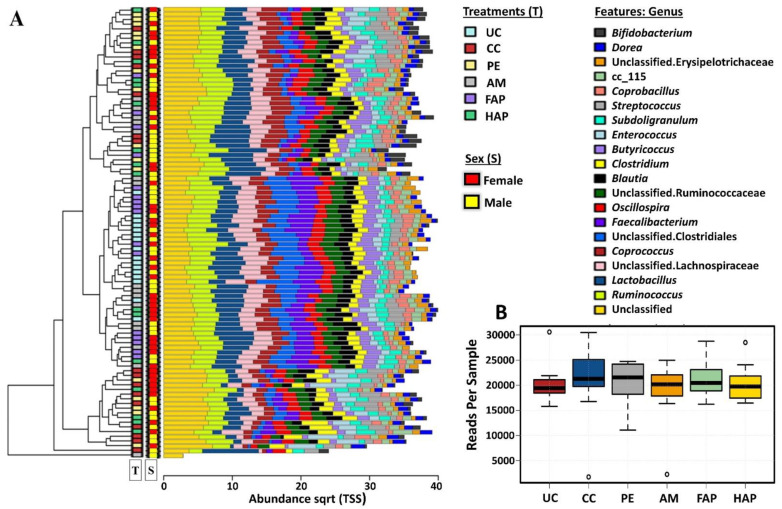
Microbial community composition on d 16 (**A**) Clustered barchart showing 20 most abundant genera and (**B**) Sequence reads per sample. UC, unchallenged control; CC, challenged control; PE, plant extract; AM, antimicrobial (narasin and nicarbazin; 50 ppm each active compound); FAP, full dose of AM plus PE; HAP, half dose of AM plus PE. ° The circle in B shows the reads of the individual sample.

**Figure 3:**
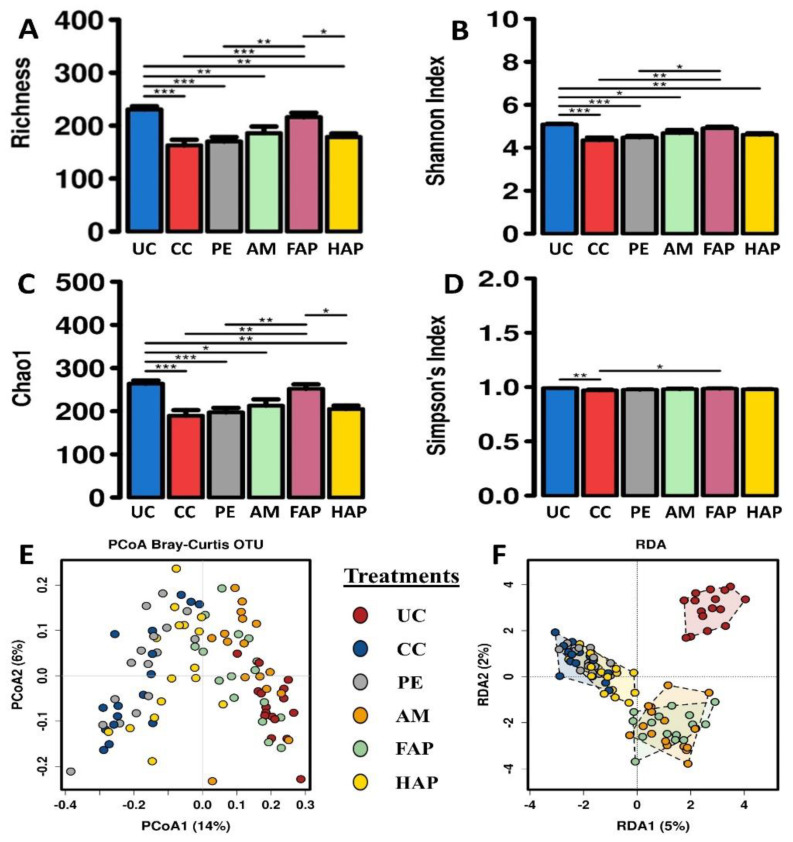
Diversity of microbiota in the caecal content on d 16 showing alpha diversity indicesindices (**A**) Richness (*p* = 7 × 10^−7^), (**B**) Shannon Index (*p* = 4.5 × 10^−6^), (**C**) Chao1 (*p* = 4.5 × 10^−6^) and (**D**) Simpson’s Index (*p* < 0.001), and beta diversity (**E**) Principal coordinate analysis and (**F**) Redundancy analysis (*p* < 0.001) at OTU level. UC, unchallenged control; CC, challenged control; PE, plant extract; AM, antimicrobial (narasin and nicarbazin; 50 ppm each active compound); FAP, full dose of AM plus PE; HAP, half dose of AM plus PE. * (*p* < 0.05),** (*p* < 0.01) and *** (*p* < 0.001).

A total of 96 caecal DNA samples from different treatment groups (male = 48 and female = 48 birds) were sequenced for this study. Microbial community composition on d 16 is shown in Figure 2A, clustered bar chart of 20 most abundant genera and sequence reads per sample in Figure 2B. There were significant differences in alpha diversity indices (Figure 3A–D) among different treatment groups as assessed by Richness (*p* = 7 × 10^−7^), Chao1 (*p* = 4.5 × 10^−6^), Shannon index (*p* = 4.5 × 10^−6^) and Simpson’s index (*p* < 0.001). There were no significant differences in alpha diversity in male and female birds as assessed by Richness (*p* = 0.91), Chao1 (*p* = 0.82), Shannon index (*p* = 0.37) and Simpson’s Index (*p* = 0.26). Alpha diversity indices showed that the NE challenge significantly decreased richness (Richness and Chao1 indexes) and evenness (Shannon and Simpson indexes) as compared between CC group and UC group. Birds fed PE had similar richness and evenness to the CC and AM groups. Birds fed FAP and HAP had similar richness and evenness to the AM group. Multivariate analysis, PCoA Bray-Curtis and RDA (*p* < 0.001) of OTU abundance were tested for visualisation (Figure 3E,F, respectively). The RDA and PCoA analysis showed that the UC group and CC were distinctly separated whereas the CC and PE were grouped closely. The PE and AM were grouped separately whereas the AM and FAP were grouped closely while the HAP was grouped in between PE and AM groups as expected.

**Figure 4:**
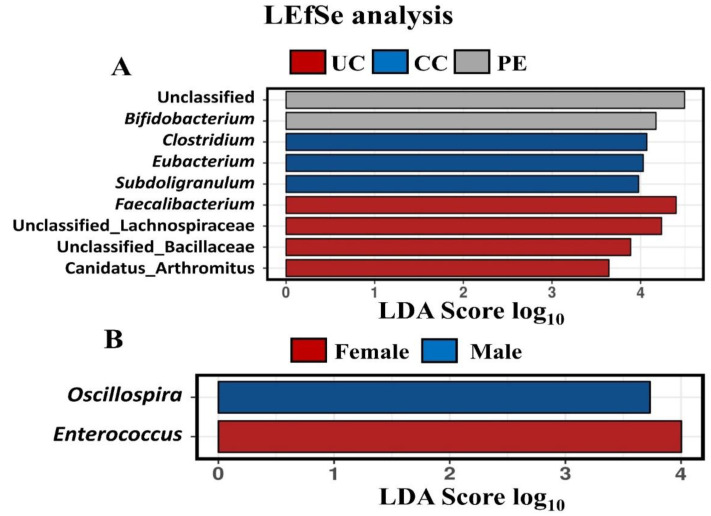
Relative abundance of caecal microbiota on d 16 showing linear discriminant analysis (LDA) effect size method (LEfSe) (**A**) treatment groups and (**B**) sex. UC, unchallenged control; CC, challenged control; PE, plant extract; AM, antimicrobial.

The microbiota community differences at the genus level were compared in different treatment groups as well as male and female birds using the linear discriminant analysis (LDA) effect size method (LEfSe, Figure 4A,B). The LEfSe analysis identified that the UC group was characterised with *Faecalibacteria*, *unclassified Lachnospiraceae*, *unclassified Bacillaceae* and *Candidatus Arthromitus*. The CC group was characterised with *Clostridium*, *Eubacterium* and *Subdoligranulum*. The PE group was identified to have the most abundance of unclassified and *Bifidobacterium* among all the groups. The male birds were characterised with *Oscillospira* and the female birds were characterised with *Enterococcus*.

**Figure 5:**
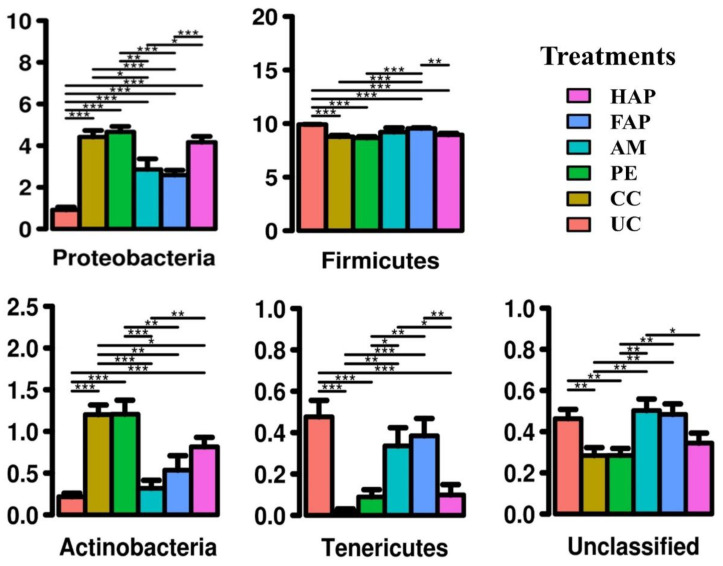
Relative abundance of caecal microbiota at phylum level on d 16. UC, unchallenged control; CC, challenged control; PE, plant extract; AM, antimicrobial (narasin and nicarbazin; 50 ppm each active compound); FAP, full dose of AM plus PE; HAP, half dose of AM plus PE. * (*p* < 0.05), ** (*p* < 0.01) and *** (*p* < 0.001).

Two-way ANOVA analysis showed a significant main effect of experimental treatment (Figure 5) on Proteobacteria, Actinobacteria, Tenericutes, Firmicutes and unclassified phyla (adjusted *p* < 0.001 for all, Bonferroni). There was no sex effect at phylum level (adjusted *p* > 0.05) and no interaction between experimental treatment and sex (*p* > 0.05). The NE challenge significantly increased the abundance of Proteobacteria and Actinobacteria, and decreased Firmicutes, Tenericutes and unclassified phyla. The abundance of bacteria at the phylum level was not different between the CC and PE groups. Birds fed PE had a similar abundance of Firmicutes compared to the AM group. Birds fed FAP had a similar abundance of bacteria at phylum level compared to the AM group, while birds fed HAP had a different abundance of Proteobacteria, Actinobacteria, Tenericutes and unclassified phyla from AM group, but a similar abundance of Firmicutes.

## 4. Discussion

The supplementation of plant extracts to some extent has shown to be effective against sub-clinical form of NE as reported in recent reviews [16,17,18]. However, the mechanism of action of plant extracts on intestinal health is still not fully understood. Moreover, for them to be potential alternatives to in-feed AM in the poultry industry, it is necessary to examine the efficacy of feed additives under severe diseased conditions. The current study assessed the effects of PE, a microencapsulated product composed of eugenol and garlic tincture on intestinal permeability, duodenal histomorphology, jejunal gene expressions, ileal and caecal microbiota of birds under a clinical NE challenge. The objective was to investigate the potential of PE supplementation in diet to modulate intestinal environment of broilers, especially under challenged conditions. The results observed in this study showed that birds supplemented with PE had reduced serum FITC-d, increased goblet cell numbers per villus, downregulated *CLDN5* gene in male birds and decreased *Bacteroides* spp. loads in female birds compared to the CC group. Furthermore, PE supplementation reduced CD, and had no differences of VW, VH:CD, goblet cell numbers per villus and alpha diversity indices compared to the AM group. These findings support the hypothesis that dietary inclusion of PE improves intestinal integrity and helps to protect against intestinal damage in birds to some extent under the severe NE challenge condition. However, contrary to our anticipations, diets supplemented with PE did not show synergistic effects with AM, as the effects of PE supplementation in combination with a full-dose AM did not demonstrate benefit compared to the birds fed AM alone. These findings reject our hypothesis that PE supplementation in combination with AM may create synergism and modulate the intestinal environment to promote intestinal health status in protecting birds against clinical NE. These findings confirm the performance results from the same experiment reported earlier [31].

In the current study, a successful clinical NE challenge was induced as shown by the evident signs of NE such as reduced body weight gain, feed intake, increased feed conversion ratio and intestinal lesions with high mortality of birds observed in the CC group compared to the UC group [31]. It has been reported that birds challenged with NE have been associated with reduced feed intake, body weight gain and increased feed conversion ratio [9,64] and increased mortality of up to 50% in severe conditions [7,8]. In the current study, birds challenged with *Eimeria* spp. and *C. perfringens* have shown significant effects on intestinal health as indicated by increased permeability, impaired histomorphology, tight junction genes and disrupts the microbial balance. These results further confirm a successful introduction of NE challenge which are in line with performance, intestinal lesions and *Eimeria* spp. oocyst counts results that have been reported earlier [31]. It should be noted that the NE challenge model employed in this study, where field strains of *Eimeria* spp. oocysts were used as a predisposing factor and *C. perfringens* as a causative agent to induce NE. Therefore, it is plausible that the inoculation of *Eimeria* spp. containing *E*. *acervulina*, *E*. *maxima* and *E*. *brunetti* prior to *C. perfringens* to succumb NE may have contributed to microbial shifts and intestinal damage observed in this study.

The results observed in the current study showed that birds fed PE had reduced pathogenic *Bacteroides* spp. in the ileal content of female birds compared to the CC group and increased the abundance of beneficial *Bifidobacterium* (LefSe analysis) in caecal content, suggesting the beneficial effects of a feed additive on small intestine against pathogens. This study showed that birds fed PE had reduced serum FITC-d compared to the CC group. This indicates the improved intestinal integrity in birds fed PE possibly by controlling *Eimeria* replication. The improvement is consistent with the observation that PE reduced ileal and caecal *Eimeria* spp. oocyst counts as reported earlier [31]. The increased goblet cell numbers per villus, beneficial bacterial loads and reduced pathogenic bacterial loads shown in this study are also shreds of evidence for the improved intestinal health of the broilers by the PE supplementation. It has been shown that enteric diseases such as NE damages mucosa and impair the function of tight junctions leading to increased intestinal permeability [10]. Furthermore, enteric inflammation can reduce goblet cell numbers, primary sites for mucin secretions that subsequently disrupt the function of mucins to act as a barrier against pathogens [65]. Thus, increased goblet cell numbers in the PE supplemented group demonstrated the beneficial effects of its supplementation in protecting mucosal damage which in turn improved intestinal integrity. Moreover, birds fed PE had similar VH:CD, goblet cell numbers and alpha diversity indices to the AM treated birds further confirming the protective effects of PE on intestinal health. Altogether, the current study data suggest beneficial effects of PE supplementation on intestinal health might have contributed to the improved feed conversion ratio, livability, uniformity, reduced ileal lesions and increased skin yellowness as reported previously [31].

The intestinal barrier functions are regulated by tight junction proteins including CLDN1, CLDN5, TJP1, JAM2 and OCLN. The disruption of tight junction protein expression leads to enteric leakage and epithelial cell damage. Epithelial cells and mucosa play a frontline defence against pathogenic invasion and help to maintain intestinal homeostasis. Thus, damages in the epithelial cells and mucosa lead to a disruption of microbial inhabitants and balance resulting in impaired nutrient absorption and performance [66,67]. The results observed in the current study showed that NE challenge downregulated *OCLN* in male birds and *MUC2* in both male and female birds. These results were consistent with previous findings in broilers where birds challenged with NE had downregulated *OCLN* genes [41]. Interestingly, *CLDN1* and *JAM2* were upregulated by NE challenge, and it is contrary to their function as tight junction proteins. However, as discussed by Gharib-Naseri et al. [41], the upregulation of *CLDN1* may not be an indication of better tight junction function but other mechanisms may be involved. Therefore, we speculate that it may not be a good marker for the indication of gut integrity at least in the NE challenge studies. The upregulation of *JAM2* is also opposite to what we expect, and further investigation is warranted to determine its response to NE challenge and possibly alternative functions. Although the expression of these tight junction and immunity genes were not different between PE and CC groups, PE supplementation led to the expression of *CLDN1*, *OCLN* and *MUC2* genes elevated to be statistically similar to the AM group. Furthermore, birds fed PE and AM had downregulated *CLDN5* gene compared to the CC group in male birds but the expression of *CLDN5* gene was not different between birds fed PE and AM. These results were consistent with previous findings in broilers where birds fed antimicrobials had downregulated *CLDN5* gene compared to the birds fed without additives under sub-clinical NE [11] and clinical NE challenged conditions [43]. These results indicate the similar effects of PE supplementation to AM implying its positive role in protecting the intestinal barrier of birds from pathogenic bacterial infection.

The intestinal microbiota is comprised of a highly complex ecosystem that directly or indirectly interacted with the host’s health and performance. The microbiota profoundly influenced the physiological, nutritional, metabolic and immunological status of the host. It is widely accepted that enteric diseases such as NE alters the microbiota composition of birds in the small intestine [4,11,68,69]. Such microbiota changes during disease outbreaks may indicate the immune-modulatory and inflammatory responses of the intestine. Therefore, it is apparent that microbiota plays a crucial role in NE occurrence and severity [70]. In this study, the caecal microbiota structure and composition were analysed via 16S rRNA sequencing to further investigate the mode of action underlying PE in alleviating NE-induced infection in the intestine. The results observed in the present study showed that NE challenge significantly decreased alpha diversity indicated by richness and evenness as also shown before Keerqin et al. [11]. In addition, PCoA and RDA analyses showed that NE challenge altered beta diversity where the CC and UC were distinctly grouped. Furthermore, NE challenge decreased Firmicutes, Tenericutes, unclassified phyla, and increased the abundance of Proteobacteria and Actinobacteria phyla., In addition, NE challenge increased the abundance of *Clostridium*, *Eubacterium* and *Subdoligranulum* at the genus level. These results together indicated the large microbial shifts in birds under NE challenge which further confirms the disruption of the intestinal environment by NE. Interestingly, PE addition did not show the positive effects in the control of *Clostridium* in this study which was also evidenced with individual bacterial quantification results by qPCR. Although bacterial populations were not different between the PE and CC groups, PE supplementation led to alpha diversity and Firmicutes phylum to the level statistically similar to the AM group. However, the increased abundance of *Bifidobacterium*, a genus where probiotics are isolated, in the PE group may indicate a positive effect of the additive to the broilers challenged with NE.

Similar to the caecal microbiota results, the NE challenge significantly affects the bacterial composition in ileal content. Birds challenged with NE had different bacterial loads compared to the UC group observed in this study. The results showed that female birds fed PE had reduced *Bacteroides* spp. loads compared to the CC group. *Bacteroides* spp. are common intestinal flora in healthy birds that, however, can turn up to be pathogenic under diseased conditions [71]. It has shown that *Bacteroides* spp. can be increased in the intestine when the birds are infected with pathogens [72]. It is likely that the increased load of *Bacteroides* spp. in the intestine is due to the higher loads *C. perfringens* and/or *Eimeria* colonisation in the intestine of the birds under NE challenge in this study. These bacteria have shown excessive immunostimulatory and proteolytic activities that negatively affect the immune response and impair intestinal health of the host [71,73]. Therefore, reduced *Bacteroides* spp. loads by the inclusion of PE in diets may indicate the beneficial effects of PE against pathogenic bacteria. However, further study is required to understand how PE supplementation suppresses the growth of *Bacteroides* spp in the gut of challenged broilers.

## 5. Conclusions

The current study indicates that the addition of a microencapsulated product composed of eugenol and garlic tincture can improve intestinal integrity and increase mucin-producing goblet cell numbers as a defensive response to the birds against NE. The dietary addition of PE also modulates microbiota balance by suppressing pathogenic while promoting beneficial microbial growth in the intestine as a secondary defensive mechanism. Moreover, it has been shown that birds fed PE had improved feed efficiency and reduced the severity of clinical NE challenge on intestinal health as indicated by reduced intestinal lesions, and ileal and caecal *Eimeria* spp. oocyst counts [31]. Therefore, it is recommended that PE supplementation could be beneficial to the NE affected broilers as the current study findings. Further research is warranted to better understand the mechanism and possible effects of PE supplementation on the birds under NE challenged conditions.

## Figures and Tables

**Table 1:** Treatments applied in this study.

Treatments ^1^	Additives	Inclusion Level; Starter (d 0 to 9), Grower (d 9 to 21) and Finisher (d 21 to 35) Phases, ppm	Necrotic Enteritis Challenge ^2^
UC	-	-	Unchallenged
CC	-	-	Challenged
PE	Plant extract	100	Challenged
AM	Antimicrobial	50 of narasin and nicarbasin	Challenged
FAP	AM full dose + PE	50 + 100	Challenged
HAP	AM half dose + PE	25 + 100	Challenged

ppm = part per million. ^1^ UC, unchallenged control; CC, challenged control; PE, plant extract; AM, antimicrobial (narasin and nicarbasin; 50 ppm each active compound); FAP, full dose of AM plus PE; HAP, half dose of AM plus PE. ^2^ Challenged birds were orally gavaged with *Eimeria* spp. on d 9 and *Clostridium perfringens* on d 14.

**Table 2:** Sequences of primers used for quantitative real-time PCR.

Item	Sequence	Size (pb)	Annealing Temperature (°C)	Reference
*TJP1*	F-GGATGTTTATTTGGGCGGC R-GTCACCGTGTGTTGTTCCCAT	187	60	Gharib-Naseri et al. [41]
*OCLN*	F-ACGGCAGCACCTACCTCAA R-GGGCGAAGAAGCAGATGAG	123	60	Du et al. [42]
*CLDN1*	F-CTTCATCATTGCAGGTCTGTCAG R-AAATCTGGTGTTAACGGGTGTG	103	60	Gharib-Naseri et al. [41]
*CLDN5*	F-GCAGGTCGCCAGAGATACAG R-CCACGAAGCCTCTCATAGCC	162	61	Kumar et al. [43]
*JAM2*	F-AGACAGGAACAGGCAGTGCTAG R-ATCCAATCCCATTTGAGGCTAC	135	60	Kumar et al. [43]
*MUC2*	F-CCCTGGAAGTAGAGGTGACTG R-TGACAAGCCATTGAAGGACA	143	60	Fan et al. [44]
Reference Genes on d 8				
*TBP*	F-TAGCCCGATGATGCCGTAT R-GTTCCCTGTGTCGCTTGC	66	61	Li et al. [45]
*YWHAZ*	F-TTGCTGCTGGAGATGACAAG R-CTTCTTGATACGCCTGTTG	61	60	Bagés et al. [46]
Reference Genes on d 16				
*GAPDH*	F: GAAGCTTACTGGAATGGCTTTCC R: CGGCAGGTCAGGTCAACAA	66	61	Kuchipudi et al. [47]
*TBP*	F-TAGCCCGATGATGCCGTAT R-GTTCCCTGTGTCGCTTGC	147	62	Li et al. [45]

TJP1 = Tight junction protein 1; OCLN = Occludin; CLDN1 = Claudin1; CLDN5 = Claudin 5; JAM2 = Junctional Adhesion Molecule 2; MUC2 = Mucin 2; TBP = TATA-Box Binding Protein; YWHAZ = Tyrosine 3-Monooxygenase/Tryptophan 5-Monooxygenase Activation Protein Zeta; GAPDH = Glyceraldehyde 3-phosphate dehydrogenase.

**Table 3:** The specific primers used for quantifying bacteria in ileal and caecal digesta.

Bacteria Group	Primer Sequence (5′→3′)	Annealing Temperature (°C)	Reference
*Lactobacillus* spp.	F-CAC CGC TAC ACA TGG AG R-AGC AGT AGG GAA TCT TCC A	63	Rinttilä et al. [52]
*Bifidobacterium* spp.	F-GCG TCC GCT GTG GGC R-CTT CTC CGG CAT GGT GTT G	63	Requena et al. [53]
*Bacteroides* spp.	F-GAG AGG AAG GTC CCC CAC R-CGC TAC TTG GCT GGT TCA G	63	Layton et al. [54]
*Bacillus* spp.	F-GCA ACG AGC GCA ACC CTT GA R-TCA TCC CCA CCT TCC TCC GGT	63	Zhang et al. [55]
*Ruminococcus* spp.	F-GGC GGC YTR CTG GGC TTT R-CCA GGT GGA TWA CTT ATT GTG TTA A	63	Ramirez-Farias et al. [56]
Enterobacteriaceae	F-CAT TGA CGT TAC CCG CAG AAG AAG C R-CTC TAC GAG ACT CAA GCT TGC	63	Bartosch et al. [57]
*Clostridium perfringens*	F-ATG CAA GTC GAG CGA KG R-TAT GCG GTA TTA ATC TYC CTT T TaqMan Probe-5′-FAM-TCA TCA TTC AAC CAA AGG AGC AAT CC-TAMRA-3′	60	Rinttilä et al. [52]; Wise and Siragusa [58]
Total Bacteria	F-CGG YCC AGA CTC CTA CGG G R-TTA CCG CGG CTG CTG GCA C	63	Lee et al. [59]

**Table 4:** Effect of PE and NE challenge on serum FITC-d (μg/mL) on day 16 ^1^.

Treatment ^2^	Serum FITC-d
Experimental treatment	
UC	0.221 ^d^
CC	0.647 ^a^
PE	0.558 ^b^
AM	0.458 ^c^
FAP	0.428 ^c^
HAP	0.548 ^b^
SEM	0.018
Sex	
Male	0.462
Female	0.491
SEM	0.011
*p*-value	
Experimental treatment	<0.001
Sex	0.064
Experimental treatment × Sex	0.502

PE = plant extract; NE = necrotic enteritis; FITC-d = fluorescein isothiocyanate dextran. ^1^ UC, unchallenged control; CC, challenged control; PE, plant extract; AM, antimicrobial (narasin and nicarbazin; 50 ppm each active compound); FAP, full dose of AM plus PE; HAP, half dose of AM plus PE. ^2^ Challenged birds were orally gavaged with *Eimeria* spp. on d 9 and *Clostridium perfringens* on d 14. ^a–d^ Values in a column with no common superscripts differ significantly (*p* < 0.05).

**Table 5:** Effects of PE and NE challenge on histomorphology in broilers on d 8 and d 16 ^1^.

Item	UC	NE Challenged ^2^					SEM	*p*-Value
		CC	PE	AM	FAP	HAP		
d 8 (before NE challenge)								
VH, μm	1471	1460	1514	1480	1523	1495	32	0.715
CD, μm	219	218	209	222	217	210	8	0.789
VW, μm	149	146	149	148	149	147	2	0.788
VH:CD	6.75	6.76	7.31	6.72	7.03	7.17	0.20	0.198
VSA, mm ^2^	0.686	0.669	0.707	0.686	0.714	0.687	0.015	0.323
Goblet cell number/villus	89.7 ^c^	90.3 ^b,c^	93.5 ^a–c^	92.7 ^a–c^	96.3 ^a^	94.9 ^a,b^	1.6	0.041
d 16 (after NE challenge)								
VH, μm	1685 ^a^	1057 ^d^	1153 ^c,d^	1306 ^b^	1316 ^b^	1290 ^b,c^	35	<0.001
CD, μm	236 ^c^	365 ^a,b^	349 ^b^	391 ^a^	396 ^a^	372 ^a,b^	14	<0.001
VW, μm	172 ^d^	212 ^c^	215 ^b,c^	230 ^a,b^	245 ^a^	222 ^b,c^	6	<0.001
VH:CD	7.23 ^a^	2.94 ^c^	3.36 ^b,c^	3.34 ^b,c^	3.33 ^b,c^	3.48 ^b^	0.17	<0.001
VSA, mm ^2^	0.909 ^a,b^	0.703 ^c^	0.780 ^b,c^	0.942 ^a^	1.015 ^a^	0.897 ^a,b^	0.032	<0.001
Goblet cell number/villus	92.4 ^a^	59.7 ^d^	68.1 ^c^	74.8 ^b,c^	77.8 ^b^	75.7 ^b,c^	2.8	<0.001

NE = necrotic enteritis; PE, plant extract; VH = villus height; CD = crypt depth; VW = villus width; VSA = vllus surface area ^1^ UC, unchallenged control; CC, challenged control; PE, plant extract; AM, antimicrobial (narasin and nicarbazin; 50 ppm each active compound); FAP, full dose of AM plus PE; HAP, half dose of AM plus PE. ^2^ Challenged birds were orally gavaged with *Eimeria* spp. on d 9 and *Clostridium perfringens* on d 14. ^a–d^ Values in a row with no common superscripts differ significantly (*p* < 0.05).

**Table 6:** Experimental treatment and sex as main effects on jejunal gene expressions before challenge (d 8) ^1^.

Treatment	*CLDN1*	*MUC2*	*OCLN*	*TJP1*
Experimental treatment				
UC	1.025	1.187	1.122	1.142
CC	1.023	1.140	1.117	1.057
PE	1.028	0.965	1.031	1.143
AM	1.057	1.145	0.989	1.027
FAP	1.075	1.063	0.976	1.091
HAP	1.133	1.096	1.187	1.074
SEM	0.094	0.111	0.096	0.109
Sex				
Male	1.015	1.121	1.155 ^a^	0.988 ^b^
Female	1.099	1.077	0.985 ^b^	1.190 ^a^
SEM	0.055	0.064	0.055	0.063
*p*-value				
Experimental treatment	0.959	0.777	0.568	0.968
Sex	0.278	0.633	0.033	0.026
Experimental treatment × Sex	0.996	0.563	0.569	0.381

^1^ UC, unchallenged control; CC, challenged control; PE, plant extract; AM, antimicrobial (narasin and nicarbazin; 50 ppm each active compound); FAP, full dose of AM plus PE; HAP, half dose of AM plus PE. ^a,b^ Values in a column with no common superscripts differ significantly (*p* < 0.05).

**Table 7:** Experimental treatment and sex as main effects on jejunal gene expressions on d 16 ^1^.

Treatment	*CLDN1*	*TJP1*	*JAM2*
Experimental treatment			
UC	0.540 ^b^	1.148	0.818 ^d^
CC	1.429 ^a^	0.986	1.198 ^a^
PE	1.312 ^a^	1.143	1.120 ^a,b^
AM	1.181 ^a^	1.163	0.893 ^c,d^
FAP	1.151 ^a^	1.154	1.008 ^b,c^
HAP	1.295 ^a^	1.157	1.096 ^a,b^
SEM	0.128	0.090	0.060
Sex			
Male	1.153	1.226 ^a^	1.050
Female	1.150	1.024 ^b^	0.989
SEM	0.074	0.052	0.035
*p*-value			
Experimental treatment	<0.001	0.721	<0.001
Sex	0.965	0.008	0.214
Experimental treatment × Sex	0.151	0.723	0.085

^1^ UC, unchallenged control; CC, challenged control; PE, plant extract; AM, antimicrobial (narasin and nicarbazin; 50 ppm each active compound); FAP, full dose of AM plus PE; HAP, half dose of AM plus PE. ^a–d^ Values in a column with no common superscripts differ significantly (*p* < 0.05). Challenged birds were orally gavaged with *Eimeria* spp. on d 9 and *Clostridium perfringens* on d 14.

**Table 8:** Interactions between experimental treatment and sex on jejunal gene expressions on d 16 ^1^.

Sex	Treatment	*CLDN5*	*OCLN*	*MUC2*
Male	UC	1.221 ^a,b^	2.191 ^a^	2.041 ^a^
	CC	1.363 ^a^	1.111 ^b–d^	0.707 ^d^
	PE	0.984 ^b–d^	1.270 ^b^	0.956 ^b–d^
	AM	0.831 ^d^	1.160 ^b,c^	1.005 ^b–d^
	FAP	0.878 ^c,d^	1.217 ^b,c^	1.186 ^b,c^
	HAP	1.039 ^a–d^	1.102 ^b–d^	1.248 ^b,c^
Female	UC	0.816 ^d^	0.999 ^b–e^	1.329 ^b^
	CC	0.990 ^b–d^	0.767 ^e^	0.870 ^c,d^
	PE	1.245 ^a,b^	0.677 ^e^	0.874 ^c,d^
	AM	0.944 ^b–d^	0.809 ^d,e^	1.080 ^b–d^
	FAP	1.280 ^a,b^	0.822 ^d,e^	1.224 ^b,c^
	HAP	1.175 ^a–c^	0.907 ^c–e^	1.018 ^b–d^
*p*-value				
Experimental treatment		0.243	<0.001	<0.001
Sex		0.750	<0.001	0.108
Experimental treatment × Sex		0.004	<0.001	0.029

^1^ UC, unchallenged control; CC, challenged control; PE, plant extract; AM, antimicrobial (narasin and nicarbazin; 50 ppm each active compound); FAP, full dose of AM plus PE; HAP, half dose of AM plus PE. ^a–e^ Values in a column with no common superscripts differ significantly (*p* < 0.05). Challenged birds were orally gavaged with *Eimeria* spp. on d 9 and *Clostridium perfringens* on d 14.

**Table 9:** Experimental treatment and sex as main effects on ileal microbiota on d 16 ^1^.

Treatment	*Lactobacillus*	*Bifidobacteria*	*Bacillus*	*Ruminococcus*	*Enterobacteriaceae*	*C. perfringens*	Total Bacteria
Experimental treatment							
UC	7.26 ^d^	5.96 ^e^	6.07 ^d^	5.69	5.30 ^c^	3.94 ^c^	8.82 ^d^
CC	8.20 ^a^	6.86 ^a^	6.81 ^a,b^	6.08	8.21 ^a^	9.16 ^a^	9.92 ^a^
PE	8.06 ^a,b^	6.74 ^a,b^	6.95 ^a^	5.68	8.11 ^a^	8.94 ^a,b^	9.75 ^a,b^
AM	7.57 ^c,d^	6.42 ^c,d^	6.63 ^b,c^	5.75	7.18 ^b^	4.99 ^c^	9.40 ^c^
FAP	7.51 ^c,d^	6.20 ^d,e^	6.58 ^c^	5.61	7.08 ^b^	5.19 ^c^	9.41 ^c^
HAP	7.72 ^b,c^	6.53 ^b,c^	6.81 ^a,b^	5.59	7.24 ^b^	7.73 ^b^	9.57 ^b,c^
SEM	0.10	0.09	0.07	0.16	0.21	0.46	0.10
Sex							
Male	7.61 ^b^	6.52	6.58 ^b^	5.35 ^b^	7.09	6.50	9.46
Female	7.83 ^a^	6.38	6.70 ^a^	6.11 ^a^	7.28	6.81	9.50
SEM	0.06	0.05	0.04	0.09	0.12	0.27	0.06
*p*-value							
Experimental treatment	<0.001	<0.001	<0.001	0.244	<0.001	<0.001	<0.001
Sex	0.008	0.070	0.039	<0.001	0.271	0.412	0.608
Experimental treatment × Sex	0.460	0.193	0.160	0.109	0.436	0.557	0.255

^1^ UC, unchallenged control; CC, challenged control; PE, plant extract; AM, antimicrobial (narasin and nicarbazin; 50 ppm each active compound); FAP, full dose of AM plus PE; HAP, half dose of AM plus PE. ^a–e^ Values in a column with no common superscripts differ significantly (*p* < 0.05). Challenged birds were orally gavaged with *Eimeria* spp. on d 9 and *Clostridium perfringens* on d 14.

**Table 10:** Experimental treatment and sex as main effects on caecal microbiota on d 16 ^1^.

Treatment	*Lactobacillus*	*Bifidobacteria*	*Bacteroides*	*Bacillus*	*Ruminococcus*	*Enterobacteriaceae*	*C. perfringens*	Total Bacteria
Experimental treatment								
UC	8.64 ^c^	8.46 ^bc^	5.85	7.64 ^a^	9.57 ^a^	8.48 ^d^	0.27 ^c^	10.79 ^b^
CC	9.41 ^a^	8.65 ^a^	6.01	6.84 ^c^	9.04 ^b^	10.62 ^a^	9.62 ^a^	11.05 ^a^
PE	9.09 ^b^	8.54 ^a,b^	5.88	7.02 ^b,c^	9.05 ^b^	10.23 ^a,b^	9.29 ^a^	10.85 ^a,b^
AM	8.41 ^c^	8.40 ^b,c^	5.78	7.20 ^b^	9.14 ^b^	9.28 ^c^	2.89 ^b^	10.64 ^b^
FAP	8.43 ^c^	8.36 ^c^	5.80	7.36 ^a,b^	9.21 ^b^	9.43 ^c^	3.87 ^b^	10.68 ^b^
HAP	8.64 ^c^	8.50 ^a–c^	5.85	7.11 ^b,c^	9.08 ^b^	9.79 ^b,c^	8.80 ^a^	10.83 ^a,b^
SEM	0.11	0.06	0.07	0.13	0.06	0.15	0.58	0.09
Sex								
Male	8.27 ^b^	8.31 ^b^	5.85	7.17	9.24 ^a^	9.59	5.44	10.71 ^b^
Female	9.27 ^a^	8.66 ^a^	5.87	7.22	9.13 ^b^	9.69	6.14	10.90 ^a^
SEM	0.06	0.03	0.04	0.07	0.04	0.09	0.33	0.05
*p*-value								
Experimental treatment	<0.001	0.007	0.242	<0.001	<0.001	<0.001	<0.001	0.018
Sex	<0.001	<0.001	0.742	0.655	0.031	0.457	0.136	0.007
Experimental treatment × Sex	0.670	0.100	0.553	0.761	0.665	0.538	0.538	0.592

^1^ UC, unchallenged control; CC, challenged control; PE, plant extract; AM, antimicrobial (narasin and nicarbazin; 50 ppm each active compound); FAP, full dose of AM plus PE; HAP, half dose of AM plus PE. ^a–c^ Values in a column with no common superscripts differ significantly (*p* < 0.05). Challenged birds were orally gavaged with *Eimeria* spp. on d 9 and *Clostridium perfringens* on d 14.

## Data Availability

The data presented in this study are available on request from the corresponding author. The 16S rRNA sequencing data is publicly available online (www.mg-rast.org (accessed on 30 June 2021); ID mgp99146).

## References

[1] Wade B., Keyburn A. (2015). The true cost of necrotic enteritis. Poult. World.

[2] Keyburn A.L., Boyce J.D., Vaz P., Bannam T.L., Ford M.E., Parker D., di Rubbo A., Rood J.I., Moore R.J. (2008). NetB, a new toxin that is associated with avian necrotic enteritis caused by *Clostridium perfringens*. PLoS Pathog..

[3] Moore R.J. (2016). Necrotic enteritis predisposing factors in broiler chickens. Avian Pathol..

[4] Wu S.-B., Stanley D., Rodgers N., Swick R.A., Moore R.J. (2014). Two necrotic enteritis predisposing factors, dietary fishmeal and *Eimeria* infection, induce large changes in the caecal microbiota of broiler chickens. Vet. Microbiol..

[5] Annett C., Viste J., Chirino-Trejo M., Classen H., Middleton D., Simko E. (2002). Necrotic enteritis: Effect of barley, wheat and corn diets on proliferation of *Clostridium perfringens* type A. Avian Pathol..

[6] Moran E.T. (2014). Intestinal events and nutritional dynamics predispose *Clostridium perfringens* virulence in broilers. Poult. Sci..

[7] Kaldhusdal M., Schneitz C., Hofshagen M., Skjerve E. (2001). Reduced incidence of *Clostridium perfringens*-associated lesions and improved performance in broiler chickens treated with normal intestinal bacteria from adult fowl. Avian Dis..

[8] Immerseel F.V., Buck J.D., Pasmans F., Huyghebaert G., Haesebrouck F., Ducatelle R. (2004). *Clostridium perfringens* in poultry: An emerging threat for animal and public health. Avian Pathol..

[9] Gharib-Naseri K., Kheravii S., Keerqin C., Morgan N., Swick R., Choct M., Wu S. (2019). Two different *Clostridium perfringens* strains produce different levels of necrotic enteritis in broiler chickens. Poult. Sci..

[10] Latorre J.D., Adhikari B., Park S.H., Teague K.D., Graham L.E., Mahaffey B.D., Baxter M.F., Hernandez-Velasco X., Kwon Y.M., Ricke S.C. (2018). Evaluation of the epithelial barrier function and ileal microbiome in an established necrotic enteritis challenge model in broiler chickens. Front. Vet. Sci..

[11] Keerqin C., Rhayat L., Zhang Z.-H., Gharib-Naseri K., Kheravii S., Devillard E., Crowley T., Wu S.-B. (2021). Probiotic *Bacillus subtilis* 29,784 improved weight gain and enhanced gut health status of broilers under necrotic enteritis condition. Poult. Sci..

[12] Kaldhusdal M., Benestad S.L., Løvland A. (2016). Epidemiologic aspects of necrotic enteritis in broiler chickens–disease occurrence and production performance. Avian Pathol..

[13] Kocher A., Choct M. (2008). Improving Broiler Chicken Performance: The Efficacy of Organic Acids, Prebiotics and Enzymes in Controlling Necrotic Enterities.

[14] Savoia D. (2012). Plant-derived antimicrobial compounds: Alternatives to antibiotics. Future Bicrobiol..

[15] Windisch W., Schedle K., Plitzner C., Kroismayr A. (2008). Use of phytogenic products as feed additives for swine and poultry. J. Anim. Sci..

[16] Diaz Carrasco J.M., Redondo L.M., Redondo E.A., Dominguez J.E., Chacana A., Fernandez Miyakawa M.E. (2016). Use of plant extracts as an effective manner to control *Clostridium perfringens* induced necrotic enteritis in poultry. BioMed. Res. Int..

[17] Adhikari P., Kiess A., Adhikari R., Jha R. (2020). An approach to alternative strategies to control avian coccidiosis and necrotic enteritis. J. Appl. Poult. Res..

[18] Lillehoj H., Liu Y., Calsamiglia S., Fernandez-Miyakawa M.E., Chi F., Cravens R.L., Oh S., Gay C.G. (2018). Phytochemicals as antibiotic alternatives to promote growth and enhance host health. Vet. Res..

[19] Brenes A., Roura E. (2010). Essential oils in poultry nutrition: Main effects and modes of action. Anim. Feed Sci. Technol..

[20] Si W., Gong J., Tsao R., Zhou T., Yu H., Poppe C., Johnson R., Du Z. (2006). Antimicrobial activity of essential oils and structurally related synthetic food additives towards selected pathogenic and beneficial gut bacteria. J. Appl. Microbiol..

[21] Si W., Ni X., Gong J., Yu H., Tsao R., Han Y., Chambers J. (2009). Antimicrobial activity of essential oils and structurally related synthetic food additives towards *Clostridium perfringens*. J. Appl. Microbiol..

[22] Applegate T., Klose V., Steiner T., Ganner A., Schatzmayr G. (2010). Probiotics and phytogenics for poultry: Myth or reality?. J. Appl. Poult. Res..

[23] Abou-Elkhair R., Gaafar K.M., Elbahy N., Helal M.A., Mahboub H.D., Sameh G. (2014). Bioactive effect of dietary supplementation with essential oils blend of oregano, thyme and garlic oils on performance of broilers infected with *Eimeria* species. Glob. Vet..

[24] Kirubakaran A., Moorthy M., Chitra R., Prabakar G. (2016). Influence of combinations of fenugreek, garlic, and black pepper powder on production traits of the broilers. Vet. World.

[25] Kim D.K., Lillehoj H.S., Lee S.H., Lillehoj E.P., Bravo D. (2013). Improved resistance to *Eimeria acervulina* infection in chickens due to dietary supplementation with garlic metabolites. Br. J. Nutr..

[26] Issa K.J., Omar J.A. (2012). Effect of garlic powder on performance and lipid profile of broilers. Open J. Anim. Sci..

[27] Giannenas I., Florou-Paneri P., Papazahariadou M., Christaki E., Botsoglou N., Spais A. (2003). Effect of dietary supplementation with oregano essential oil on performance of broilers after experimental infection with *Eimeria tenella*. Arch. Anim. Nutr..

[28] Sidiropoulou E., Skoufos I., Marugan-Hernandez V., Giannenas I., Bonos E., Aguiar-Martins K., Lazari D., Blake D., Tzora A. (2020). In vitro anticoccidial study of oregano and garlic essential oils and effects on growth performance, faecal oocyst output and intestinal microbiota in vivo. Front. Vet. Sci..

[29] Pirgozliev V., Rose S., Catherine I., Blanchard A. Phytogenic feed additives can alleviate the negative impact of necrotic enteritis in broilers. Proceedings of the 6th International Conference on Poultry Intestinal Health.

[30] NHMRC (2013). Australian Code for the Care and Use of Animals for Scientific Purposes.

[31] Kumar A., Sharma N.K., Kheravii S.K., Keerqin C., Ionescu C., Blanchard A., Wu S.-B. (2021). Potential of a microencapsulated mixture of eugenol and garlic tincture to improve performance and intestinal health in broilers under necrotic enteritis challenge. Anim. Nutr..

[32] England A., Kheravii S., Musigwa S., Kumar A., Daneshmand A., Sharma N., Gharib-Naseri K., Wu S. (2020). Sexing chickens (Gallus gallus domesticus) with high resolution melting analysis using feather crude DNA. Poult. Sci..

[33] Cobb500 (2018). Cobb Broiler Management Guide.

[34] Cobb500 (2018). Broiler Performance and Nutrition Supplement.

[35] Rodgers N.J., Swick R.A., Geier M.S., Moore R.J., Choct M., Wu S.-B. (2015). A multifactorial analysis of the extent to which *Eimeria* and fishmeal predispose broiler chickens to necrotic enteritis. Avian Dis..

[36] Golder H., Geier M., Forder R., Hynd P., Hughes R. (2011). Effects of necrotic enteritis challenge on intestinal micro-architecture and mucin profile. Br. Poult. Sci..

[37] Sakamoto K., Hirose H., Onizuka A., Hayashi M., Futamura N., Kawamura Y., Ezaki T. (2000). Quantitative study of changes in intestinal morphology and mucus gel on total parenteral nutrition in rats. J. Surg. Res..

[38] Samiullah S., Roberts J., Wu S.-B. (2017). Downregulation of ALAS1 by nicarbazin treatment underlies the reduced synthesis of protoporphyrin IX in shell gland of laying hens. Sci. Rep..

[39] Hellemans J., Mortier G., de Paepe A., Speleman F., Vandesompele J. (2007). qBase relative quantification framework and software for management and automated analysis of real-time quantitative PCR data. Genome Biol..

[40] Vandesompele J., de Preter K., Pattyn F., Poppe B., van Roy N., de Paepe A., Speleman F. (2002). Accurate normalization of real-time quantitative RT-PCR data by geometric averaging of multiple internal control genes. Genome Biol..

[41] Gharib-Naseri K., Kheravii S., Keerqin C., Swick R.A., Choct M., Wu S.-B. (2021). Differential expression of intestinal genes in necrotic enteritis challenged broiler chickens with two different *Clostridium perfringens* strains. Poult. Sci..

[42] Du E., Wang W., Gan L., Li Z., Guo S., Guo Y. (2016). Effects of thymol and carvacrol supplementation on intestinal integrity and immune responses of broiler chickens challenged with *Clostridium perfringens*. J. Anim. Sci. Biotechnol..

[43] Kumar A., Kheravii S.K., Li L., Wu S.-B. (2021). Monoglycerides blend reduces mortality, improves nutrient digestibility and intestinal health in broilers subjected to clinical necrotic enteritis challenge. Animals.

[44] Fan X., Liu S., Liu G., Zhao J., Jiao H., Wang X., Song Z., Lin H. (2015). Vitamin a deficiency impairs mucin expression and suppresses the mucosal immune function of the respiratory tract in chicks. PLoS ONE.

[45] Li Y.P., Bang D.D., Handberg K.J., Jorgensen P.H., Zhang M.F. (2005). Evaluation of the suitability of six host genes as internal control in real-time RT-PCR assays in chicken embryo cell cultures infected with infectious bursal disease virus. Vet. Microbiol..

[46] Bagés S., Estany J., Tor M., Pena R. (2015). Investigating reference genes for quantitative real-time PCR analysis across four chicken tissues. Gene.

[47] Kuchipudi S.V., Tellabati M., Nelli R.K., White G.A., Perez B.B., Sebastian S., Slomka M.J., Brookes S.M., Brown I.H., Dunham S.P. (2012). 18S rRNA is a reliable normalisation gene for real time PCR based on influenza virus infected cells. Virol. J..

[48] Kheravii S., Swick R.A., Choct M., Wu S.-B. (2017). Dietary sugarcane bagasse and coarse particle size of corn are beneficial to performance and gizzard development in broilers fed normal and high sodium diets. Poult. Sci..

[49] Kumar A., Toghyani M., Kheravii S.K., Pineda L., Han Y., Swick R.A., Wu S.-B. (2021). Organic acid blends improve intestinal integrity, modulate short-chain fatty acids profiles and alter microbiota of broilers under necrotic enteritis challenge. Anim. Nutr..

[50] Wise M.G., Siragusa G.R. (2007). Quantitative analysis of the intestinal bacterial community in one- to three-week-old commercially reared broiler chickens fed conventional or antibiotic-free vegetable-based diets. J. Appl. Microbiol..

[51] Kheravii S.K., Swick R.A., Choct M., Wu S.B. (2017). Coarse particle inclusion and lignocellulose-rich fiber addition in feed benefit performance and health of broiler chickens. Poult. Sci..

[52] Rinttilä T., Kassinen A., Malinen E., Krogius L., Palva A. (2004). Development of an extensive set of 16S rDNA-targeted primers for quantification of pathogenic and indigenous bacteria in faecal samples by real-time PCR. J. Appl. Microbiol..

[53] Requena T., Burton J., Matsuki T., Munro K., Simon M.A., Tanaka R., Watanabe K., Tannock G.W. (2002). Identification, detection, and enumeration of human *Bifidobacterium species* by PCR targeting the transaldolase gene. Appl. Environ. Microbiol..

[54] Layton A., McKay L., Williams D., Garrett V., Gentry R., Sayler G. (2006). Development of *Bacteroides* 16S rRNA gene TaqMan-based real-time PCR assays for estimation of total, human, and bovine fecal pollution in water. Appl. Environ. Microbiol..

[55] Zhang Y., Chen D., Yu B., He J., Yu J., Mao X., Wang J., Luo J., Huang Z., Cheng G. (2015). Spray-dried chicken plasma improves intestinal digestive function and regulates intestinal selected microflora in weaning piglets. J. Anim. Sci..

[56] Ramirez-Farias C., Slezak K., Fuller Z., Duncan A., Holtrop G., Louis P. (2008). Effect of inulin on the human gut microbiota: Stimulation of *Bifidobacterium adolescentis* and *Faecalibacterium prausnitzii*. Br. J. Nutr..

[57] Bartosch S., Fite A., Macfarlane G.T., McMurdo M.E. (2004). Characterization of bacterial communities in feces from healthy elderly volunteers and hospitalized elderly patients by using real-time PCR and effects of antibiotic treatment on the fecal microbiota. Appl. Environ. Microbiol..

[58] Wise M.G., Siragusa G.R. (2005). Quantitative detection of *Clostridium perfringens* in the broiler fowl gastrointestinal tract by real-time PCR. Appl. Environ. Microbiol..

[59] Lee D.-H., Zo Y.-G., Kim S.-J. (1996). Nonradioactive method to study genetic profiles of natural bacterial communities by PCR-single-strand-conformation polymorphism. Appl. Environ. Microbiol..

[60] Andrews S. (2010). FastQC: A Quality Control Tool for High Throughput Sequence Data.

[61] Bolyen E., Rideout J.R., Dillon M.R., Bokulich N.A., Abnet C.C., Al-Ghalith G.A., Alexander H., Alm E.J., Arumugam M., Asnicar F. (2019). Reproducible, interactive, scalable and extensible microbiome data science using QIIME 2. Nat. Biotechnol..

[62] Callahan B.J., McMurdie P.J., Rosen M.J., Han A.W., Johnson A.J., Holmes S.P. (2015). DADA2: High resolution sample inference from amplicon data. bioRxiv.

[63] Zakrzewski M., Proietti C., Ellis J.J., Hasan S., Brion M.-J., Berger B., Krause L. (2017). Calypso: A user-friendly web-server for mining and visualizing microbiome–environment interactions. Bioinformation.

[64] Timbermont L., Haesebrouck F., Ducatelle R., Van Immerseel F. (2011). Necrotic enteritis in broilers: An updated review on the pathogenesis. Avian Pathol..

[65] Mack D.R., Michail S., Wei S., McDougall L., Hollingsworth M.A. (1999). Probiotics inhibit enteropathogenic *E. coli* adherence in vitro by inducing intestinal mucin gene expression. Am. J. Physiol. Gastrointest. Liver.

[66] Ritzi M.M., Abdelrahman W., Mohnl M., Dalloul R.A. (2014). Effects of probiotics and application methods on performance and response of broiler chickens to an *Eimeria* challenge. Poult. Sci..

[67] Sánchez de Medina F., Romero-Calvo I., Mascaraque C., Martínez-Augustin O. (2014). Intestinal inflammation and mucosal barrier function. Inflamm. Bowel Dis. Ther..

[68] Stanley D., Wu S.-B., Rodgers N., Swick R.A., Moore R.J. (2014). Differential responses of cecal microbiota to fishmeal, *Eimeria* and *Clostridium perfringens* in a necrotic enteritis challenge model in chickens. PLoS ONE.

[69] Wu S.-B., Rodgers N.J., Cui G., Sun Y., Choct M. (2016). Dynamics of intestinal metabolites and morphology in response to necrotic enteritis challenge in broiler chickens. Avian Pathol..

[70] Antonissen G., Eeckhaut V., Van Driessche K., Onrust L., Haesebrouck F., Ducatelle R., Moore R.J., Van Immerseel F. (2016). Microbial shifts associated with necrotic enteritis. Avian Pathol..

[71] Wexler H.M. (2007). Bacteroides: The good, the bad, and the nitty-gritty. Clin. Microbiol. Rev..

[72] Phong S., Shanmugavelu S., Thayalini K., Noraini S., Wong H. (2010). Detection of *Lactobacillus*, *Bacteroides* and *Clostridium perfringens* in the gastrointestinal contents of chicken fed different diets by real-time PCR. J. Trop. Agric. Food Sci..

[73] Kleessen B., Kroesen A., Buhr H., Blaut M. (2002). Mucosal and invading bacteria in patients with inflammatory bowel disease compared with controls. Scand. J. Gastroenterol..

